# Advanced squamous cell carcinoma involving both upper and lower lips and oral commissure with simultaneous reconstruction by local flap: a case report

**DOI:** 10.1186/1752-1947-6-23

**Published:** 2012-01-18

**Authors:** Chairat Burusapat, Anont Pitiseree

**Affiliations:** 1 Division of Plastic and Reconstructive Surgery, Department of Surgery, Phramongkutklao Hospital, Bangkok, Thailand 10400

## Abstract

**Introduction:**

Squamous cell carcinoma is one of the most common malignant tumors of the skin and oral mucosa. However, squamous cell carcinoma involving near total upper and lower lip and oral commissure is rarely seen in the English literature. Simultaneous reconstruction of the upper and lower lips has been inconclusive and presents a challenge to the surgeon. We report such a case and outline our simultaneous reconstruction with local flaps. To the best of our knowledge this has never been reported.

**Case presentation:**

A 73-year-old Thai woman presented with a large rapidly growing squamous cell carcinoma involving the upper lip, lower lip, left oral commissure and left cheek. En bloc resection of upper lip, lower lip, left oral commissure and buccal region was performed. Left radical neck dissection and right modified neck dissection were performed. Reconstruction of the upper lip with a left nasolabial-cheek cervicofacial rotational-advancement flap and right cheek advancement with perialar crescent flap was performed. The lower lip was reconstructed with bilateral labiomental advancement flaps.

**Conclusions:**

Squamous cell carcinoma can grow rapidly and spread along the orbicularis oris muscle and across the oral commissure to the opposite lip. In advanced cancer, multimodal treatment is necessary. No gold standard in the reconstruction of both upper and lower lips has been established. We report the case of an advanced squamous cell carcinoma involving both the upper lip, lower lip, left oral commissure and buccal area and simultaneous reconstruction with local flap coverage that, to the best of our knowledge, has never been reported.

## Introduction

Squamous cell carcinoma (SCC) is one of the most common malignant tumors of the skin and oral mucosa. SCC of the lips accounts for approximately 30% of oral cavity malignancies.

The majority of these occur on the lower lip because of its great exposure to precipitating factors. SCC grows along the mucosal surfaces and infiltrates deeper structures in a predictable pattern. Tumors can spread by direct penetration, tracking along nerve and vascular invasion routes [1]. SCC of the lower lip often invades the deep muscle and mandible. SCC involving near total upper and lower lips and the oral commissure is rarely reported in the English literature. Reconstruction of both upper and lower lips has been inconclusive and presents a challenge to the surgeon. We report such a case and outline our simultaneous reconstruction with local flaps which has never been reported.

## Case presentation

**Figure 1:**
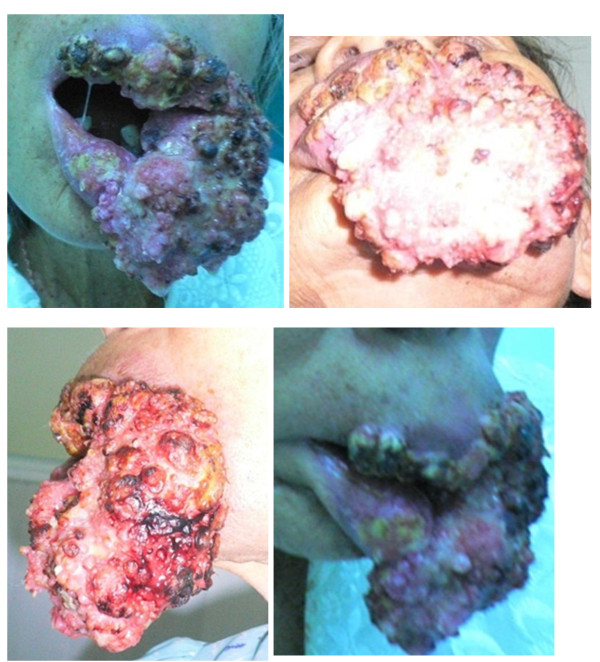
**Pre-operative view (frontal view, worm eye view, left and right lateral facial view)**.

A 73 -year-old Thai woman presented with a large rapidly growing, proliferative mass involving her upper lip, lower lip, left oral commissure and left cheek. She had a history of a small proliferative mass on the left lower lip four years previously, a total excisional biopsy that showed chronic inflammation. No residual mass was seen after that. Four months prior to admission, she had a proliferative mass on the left lower lip that rapidly grew to involve the circumoral region and the left buccal mucosa (Figure 1).

**Figure 2:**
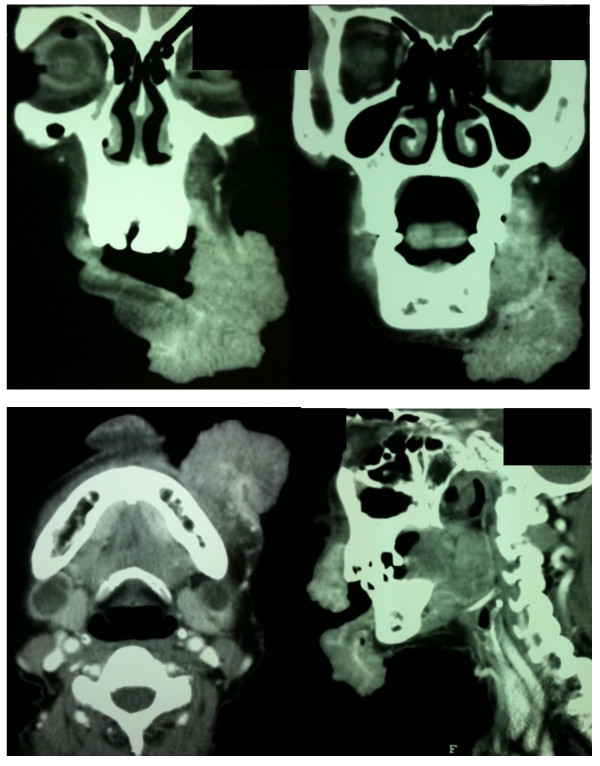
**Head and neck computed tomography (CT) examination of patient**. This CT scan showed irregular lobulated contour, heterogenous enhancing mass involving upper and lower lip (coronal view, axial view and sagittal view).

Incisional biopsy revealed well-differentiated squamous cell carcinoma. Physical examination revealed a tumor mass that involved 90% of the upper lip, 80% of the lower lip, the left oral commissure and the left buccal region including mucosa. An enlarged left submental lymph node was palpable. Sensation along her mental nerve was intact. Panorex X-ray showed no bony destruction. A computed tomography (CT) scan revealed an irregular lobulated contour, heterogeneous enhancing mass involving her upper and lower lips, predominantly on the left side but crossing the midline to the right side of the upper lip (Figure 2). The mass extended to her left buccal space, the anterior portion of the left masticator space and obliterated the fat plane of the anterior aspect of the left masseter muscle. There were necrotic submandibular lymph nodes which measured 1.4 cm. on the right and 1 cm. on the left. A CT scan also revealed multiple subcentimeter lymph nodes on both cervical regions. No definite bony invasion was observed. Her parotid glands showed no abnormality. No abnormalities were detected by a metastatic work-up. Thus, the clinical staging was T4N2cM0.

**Figure 3:**
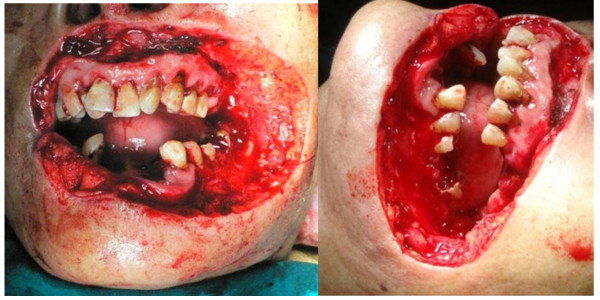
**Intra-operative after en bloc resection (frontal and lateral view)**.

En bloc resection with 1 cm margins was performed including near total upper lip (extended to both nostril sills), near total lower lip, left commissure and left buccal region. Only 0.5 cm of the upper lip and 1 cm of the lower lip could be preserved (Figure 3). In addition, left radical neck dissection and right modified neck dissection (preserved internal jugular vein and spinal accessory nerve) were performed by McFee incision.

**Figure 4:**
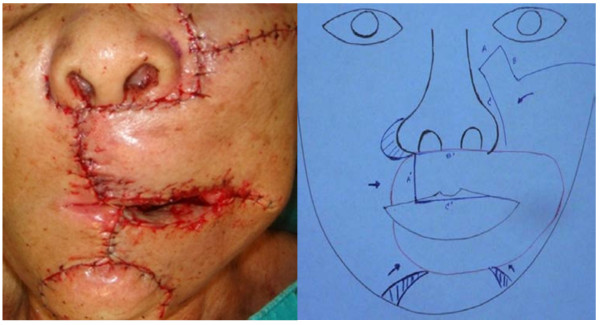
**Immediate post-operative view after simultaneous reconstruction of both upper and lower lips and diagram showed local flaps coverage**. Illustration shows left nasolabial-cheek cervicofacial rotational-advancement flap (**A-A'**, **B-B' **and **C-C'**), right cheek advancement with perialar crescent flap. Reconstruction of the lower lip with bilateral labiomental advancement flap (shaded areas were excised) (Red line shows area en bloc resection).

Examination of frozen sections revealed no malignancy in any of the surgical margins. The upper lip was reconstructed with a left nasolabial-cheek cervicofacial rotational - advancement flap and a right cheek advancement with perialar crescentic flaps. The lower lip was reconstructed with bilateral labiomental advancement flaps. Internal lining of the left buccal area was achieved with skin grafts (Figure 4).

**Figure 5:**
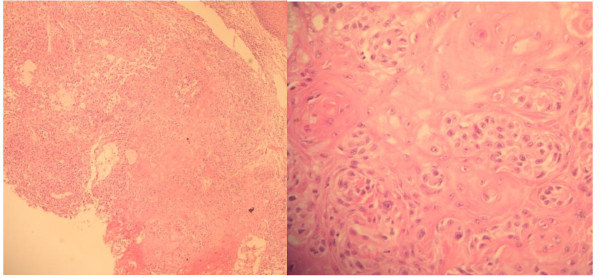
**Histopathological study of a tissue specimen taken from the lower lip**. These slides show a solid nest of polygonal cells with abundant bright-eosinophilic cytoplasm, many keratin pearls, intact intercellular bridges, few mitotic figures, and modest pleomorphic nuclei. (H&E stain, ×100 and ×400).

**Figure 6:**
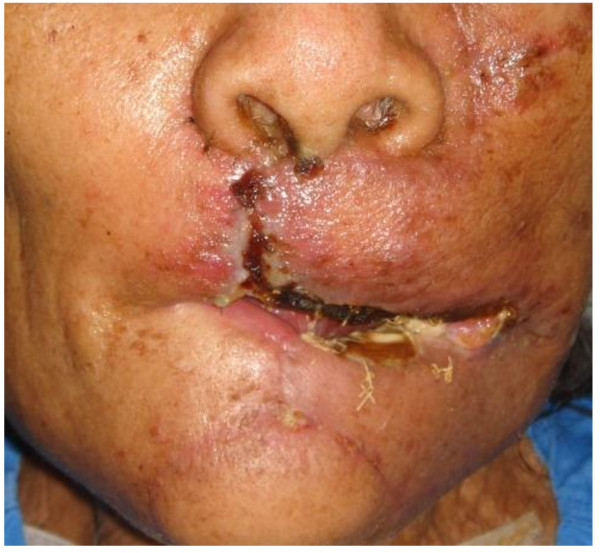
**Two weeks post-operative**.

The pathology report showed well differentiated squamous cell carcinoma with lymphovascular invasion (Figure 5). All surgical resection margins showed no malignant cells. Metastatic squamous cell carcinoma was present in two of seven left cervical lymph nodes with extracapsular extension and one of 18 right cervical lymph nodes. She developed complications of microstomia and partial left nasolabial-cheek cervicofacial flap necrosis. Only the inferior border of the upper flap became necrotic and it healed with dressings (Figure 6). She was subsequently treated with radiation commencing two weeks after surgery. External radiotherapy was performed in the fields of her primary cancer and bilateral neck. She refused chemotherapy. She could eat a soft diet with occasional mild drooling of saliva.

**Figure 7:**
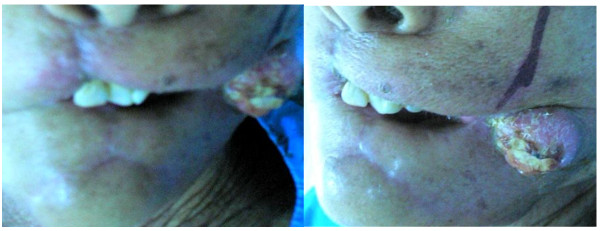
**Five months post-operative with tumor recurrence on left cheek**.

Five months later, the patient presented with an ulcerative mass at her left cheek, left axillary lymph node and dyspnea (Figure 7). Incisional biopsy of the left cheek revealed a recurrence of squamous cell carcinoma and excisional biopsy of her left axillary lymph node revealed metastatic squamous cell carcinoma. A chest X-ray showed left pleural effusion with multiple lung nodules. She died one month later due to respiratory failure.

## Discussion

SCC can be treated with surgery, radiotherapy or a combination of procedures. In advanced cancer, multimodal treatment is necessary. Early postoperative adjuvant radiation showed excellent results. SCC of the lips should be treated with surgery and postoperative radiation when there are poor prognostic indicators that include multiple levels of positive lymph nodes, extracapsular extension of the cancer in lymph nodes, deep invasion of the primary tumor, neural and vascular invasion, tumor margins less than 5 mm, and a need to take multiple layers of frozen section before a 'clear' margin is obtained [1]. Lymphadenectomy is indicated for clinically palpable lymph nodes or if a biopsy of a lymph node is positive for malignancy [2]. SCC of the lower lip may be spread along the mandible and inferior alveolar nerve [3]. In this case, although the pathology report revealed a well-differentiated lesion, the tumor grew rapidly and spread along the orbicularis oris muscle and across the oral commissure to the opposite lip without mandible or mental nerve invasion.

SCC of the upper lip and oral commissure may drain into the periparotid node so the parotid gland should be examined carefully. Patients with well-differentiated tumors have a metastatic rate of 5% but the most predictive factors for metastasis are large tumor size and high grade tumor [4]. Cervical lymph node metastasis also was the prognostic factor for lower survival rate that correlated with the outcome for this patient who had local recurrence and lung metastasis at five months after surgery. In advanced- stage cancer, pre-operative chemo-radiation is another option but this case was a resectable case, so we preferred operation and post-operative chemo-radiation.

Defects after surgery of the lips may be classified into upper lip, lower lip, and oral commissure. SCC of the lip usually involves the upper or lower lip and oral commissure, but the behavior of SCC in this case was different. The defects in this case involved three parts of the lip, reconstruction was a challenge and there is no gold standard. Reconstructive lip surgery aims to restore function, obtain a watertight-seal, maintain sensation and avoid cosmetic deformity. Many methods are available for the reconstruction of lip defects such as the Wexler and Dingman flap [5], the temporal frontal scalp flap [6] for upper lip defects and the 'staircase' technique [7], and the Schuchardt [8], Karapandzic [9], Bernard [10], Webster techniques [11] for lower lip defects. The principle of lip defects involves reconstruction with the remaining or opposite lip but there are no existing studies that describe simultaneous reconstruction of both upper and lower lips. In large defects involving both upper and lower lips, it is difficult to achieve all the goals of lip reconstruction but we desired to achieve both an oncologic and reconstructive outcome.

Reconstruction of both upper and lower lips is extremely rare and presents a challenge to the surgeon. Upper and lower lip reconstruction using microsurgery has been reported. Jallali and Malata reported reconstruction in cases of total loss of the upper and lower lips with a free vertical rectus abdominis flap in patients with fulminant pneumococcal septicemia after unsuccessful reconstruction of the upper lip with Webster's perialar advancement flap [12]. Daya reported on the reconstruction of the upper and lower lips with free radial forearm-palmaris longus tendon and brachioradialis chimeric flap in NOMA disease [13]. Nthumba and Carter reported the simultaneous reconstruction of both upper and lower lips in NOMA by using a combination of platysma flaps and deltopectoral flaps and provided mucosal lining and a scalp visor flap for both upper and lower lips [14].

In our case, we used a local flap in the reconstruction of both upper and lower lips and oral commissure in a patient with SCC, a technique that has never been reported. Although, in advanced stages of SCC, microsurgery is recommended and popular, no 'gold standard' exists for large reconstructions of both upper and lower lips, especially in SCC. Local flaps still provide a predictable method to reconstruct perioral defects following resection for oral cancer [15]. We suggest this method as another option following the step ladder of reconstruction. The main drawback of this technique is the soft tissue volume that may shrink after radiation therapy. However, we believed that the advantages of this technique are one-stage operation, less donor site morbidity and less surgical time.

Complications included partial flap necrosis and microstomia. The partial upper lip flap necrosis healed spontaneously without further surgery. In microstomia, we planned to perform a right commissuroplasty after completion of adjuvant radiation but our patient developed tumor recurrence. Although, we could not save this patient, the patient had a better quality of life.

## Conclusion

SCC is one of the most common malignant tumors but SCC involving near total upper lip, lower lip and oral commissure is rarely seen. In our patient SCC grew along the mucosal surfaces and the tumor could spread along the orbicularis oris muscle. Few reports have been published regarding simultaneous reconstruction of both upper and lower lips. Therefore, we report advanced SCC involving both upper lip, lower lip, left commissure and extending to the left buccal area treated with simultaneous resection and reconstruction with local flaps, a technique that has never been reported.

## Consent

Written informed consent was obtained from the patient for the publication of this case report and any accompanying images. A copy of the written consent is available for review by the Editor-in-Chief of this journal.

## Competing interests

The authors declare that they have no competing interests.

## Authors' contributions

CB and AP were involved in the care of the patient. CB and AP analyzed the case history, investigation and treatment. CB was the major contributor in writing the manuscript. Both authors read and approved the final manuscript.
